# Clinical features of anthracycline‐induced cardiotoxicity in patients with malignant lymphoma who received a CHOP regimen with or without rituximab: A single‐center, retrospective observational study

**DOI:** 10.1002/jha2.110

**Published:** 2020-10-03

**Authors:** Takafumi Nakayama, Yoshiko Oshima, Shigeru Kusumoto, Junki Yamamoto, Satoshi Osaga, Haruna Fujinami, Takaki Kikuchi, Tomotaka Suzuki, Haruhito Totani, Shiori Kinoshita, Tomoko Narita, Asahi Ito, Masaki Ri, Hirokazu Komatsu, Kazuaki Wakami, Toshihiko Goto, Tomonori Sugiura, Yoshihiro Seo, Nobuyuki Ohte, Shinsuke Iida

**Affiliations:** ^1^ Department of Cardiology Nagoya City University Graduate School of Medical Sciences Nagoya Japan; ^2^ Department of Hematology and Oncology Nagoya City University Graduate School of Medical Sciences Nagoya Japan; ^3^ Clinical Research Management Center Nagoya City University Hospital Nagoya Japan

**Keywords:** anthracycline‐induced cardiotoxicity, CHOP regimen, malignant lymphoma, re‐cardiotoxicity, recovery from cardiotoxicity

## Abstract

We investigated the incidence of cardiotoxicity, its risk factors, and the clinical course of cardiac function in patients with malignant lymphoma (ML) who received a cyclophosphamide, doxorubicin, vincristine, and prednisolone (CHOP) regimen. Among all ML patients who received a CHOP regimen with or without rituximab from January 2008 to December 2017 in Nagoya City University hospital, 229 patients who underwent both baseline and follow‐up echocardiography and had baseline left ventricular ejection fraction (LVEF) ≥50% were analyzed, retrospectively. Cardiotoxicity was defined as a ≥10% decline in LVEF and LVEF < 50%; recovery from cardiotoxicity was defined as a ≥5% increase in LVEF and LVEF ≥50%. Re‐cardiotoxicity was defined as meeting the criteria of cardiotoxicity again. With a median follow‐up of 1132 days, cardiotoxicity, symptomatic heart failure, and cardiovascular death were observed in 48 (21%), 30 (13%), and 5 (2%) patients, respectively. Multivariate analysis demonstrated that history of ischemic heart disease (hazard ratio (HR), 3.15; 95% CI, 1.17‐8.47, *P* = .023) and decreased baseline LVEF (HR per 10% increase, 2.55; 95% CI, 1.49‐4.06; *P* < .001) were independent risk factors for cardiotoxicity. Recovery from cardiotoxicity and re‐cardiotoxicity were observed in 21 of 48, and six of 21, respectively. Cardiac condition before chemotherapy seemed to be most relevant for developing cardiotoxicity. Furthermore, Continuous management must be required in patients with cardiotoxicity, even after LVEF recovery.

## 1 INTRODUCTION

Anthracycline is recognized as an effective chemotherapeutic agent for several kinds of tumor. Therefore, its cardiotoxicity, which can disturb chemotherapy, is currently the subject of crucial discussion [1, 2, 3, 4, 5, 6, 7, 8, 9, 10, 11, 12]. On the basis of convincing evidence proving anthracycline's benefits for malignant lymphoma (ML) and other cancers [7, 13, 14], many clinicians continue to use this agent, so an adequate understanding of this adverse effect and a plan to counter it are needed. After several risk factors for cardiotoxicity such as high cumulative dose of anthracycline and general cardiovascular risk factors were confirmed [3, 12], clinicians have tended to avoid using over 400 mg/m^2^, and recently over 250 mg/m^2^, of anthracycline [15], and have begun total cardiovascular care for patients who receive anthracycline‐containing chemotherapy. Furthermore, strategies to prevent cardiotoxicity have been studied frequently and have shown convincingly that angiotensin‐converting enzyme inhibitors, beta blockers, mineralocorticoid receptor antagonists, and statins are relevant to the prevention of cardiotoxicity [16, 17, 18, 19]. According to a recent study, anthracycline‐induced cardiotoxicity appears within 1 year after chemotherapy starts, and half or more of these cardiotoxicities are reversible to the normal range of left ventricular ejection fraction (LVEF) [20]. These findings are very important, as the number of cancer survivors is growing. However, the detailed clinical features and risk factors related to anthracycline‐induced cardiotoxicity for each disease or chemotherapy regimen have not been clarified. Particularly, there is only limited evidence regarding anthracycline‐induced cardiotoxicity in patients with ML who receive cyclophosphamide, doxorubicin, vincristine, and prednisolone (CHOP) as a uniform chemotherapy regimen. The aim of the current study was to clarify the risk factors, clinical features, and prognosis of anthracycline‐induced cardiotoxicity in patients with ML who received a CHOP regimen with or without anti‐CD20 antibody, rituximab.

## 2 METHODS

### 2.1 Study population and design

**FIGURE 1:**
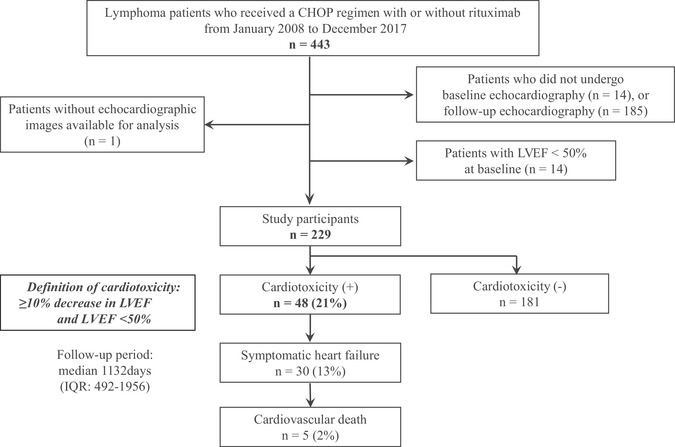
Study flow diagram including the steps from the screened patients to the finally analyzed patients, and the incidence of cardiotoxicity and other cardiac events. Two hundred twenty‐nine patients who underwent baseline and at least one or more follow‐up echocardiography studies were available for analysis, and those with baseline LVEF ≥50% were eligible for the current study. With a median follow‐up period of 1132 days, forty‐eight patients (22%) had cardiotoxicity. Of them, 30 patients (13%) experienced symptomatic heart failure and five patients (2%) died from cardiovascular causes

All 443 patients with ML who received a CHOP regimen with or without rituximab from January 2008 to December 2017 in Nagoya City University hospital were retrospectively reviewed. Of these, 229 patients with LVEF ≥ 50% at baseline who underwent baseline and at least one or more follow‐up echocardiography studies were enrolled (Figure 1).

We collected each patient's clinical data (such as age, sex, height, body weight, blood pressure, heart rate, past history, oral medication, and so on), laboratory data (white blood cell count, hemoglobin, platelet count, total protein, albumin, C‐reactive protein, aspartate transaminase, alanine transaminase, lactic acid dehydrogenase, serum creatinine, triglyceride, low‐density lipoprotein cholesterol, serum sodium, potassium, total bilirubin and brain natriuretic peptide [BNP]), and echocardiographic findings (LVEF and findings of valvular heart disease) from electronic health records in our hospital.

Two hematologists (Y.O. and S.K.) confirmed the underlying primary disease, chemotherapeutic regimen, doses of agents, and patient's performance status. Patients with intravascular large B‐cell lymphoma (IVL) and primary mediastinal large B cell lymphoma (PMLBCL) were enrolled as a subtype of diffuse large B‐cell lymphoma (DLBCL) in the current study. Two cardiologists (T.N. and J.Y.) assessed the echocardiographic data and incidences of symptomatic heart failure and cardiovascular death, which were independently evaluated in a blind manner.

Cardiotoxicity was defined as a 10% or greater decrease in LVEF and absolute LVEF < 50% by follow‐up echocardiography after chemotherapy [20]. Partial recovery was defined as a 5% or more increase in LVEF and LVEF > 50% after cardiotoxicity occurred, and full recovery was defined as improving to 95% or more of baseline LVEF. Re‐cardiotoxicity was defined as meeting the criteria of cardiotoxicity again once partial recovery or full recovery was achieved.

Cardiovascular risk factors were defined as hypertension, dyslipidemia, diabetes mellitus, and smoking. Hypertension was defined as a record of history of hypertension or being on antihypertensive treatment. Hyperlipidemia was defined as a low‐density lipoprotein‐cholesterol level of ≥140 mg/dL or being on cholesterol‐lowering treatment. Diabetes mellitus was defined as an HgA1c level of ≥6.5% or receiving treatment with blood glucose‐lowering medicine. We recorded the past histories of ischemic heart disease that included myocardial infarction, angina pectoris and history of coronary artery revascularization, heart failure, atrial fibrillation, and cerebral infarction of each patient from their medical records.

This retrospective study was approved by the Institutional Review Board of Nagoya City University and was carried out in accordance with the principles of the Helsinki Declaration.

### 2.2 Echocardiography

LVEF was measured using the disk summation method, using a TomTec imaging system (TomTec Imaging System GmbH, Munich, Germany) by cardiologists (T.N. or J.Y.). Echocardiographic data were collected according to the recommendations of the American Society of Echocardiography [21]. Valvular diseases were graded according to the guidelines of the American Heart Association [22]. To avoid a misdiagnosis of cardiotoxicity and recovery from diagnosis, we re‐measured LVEF that was between 40% and 60% by the first measurer, by the second measurer (the other of the first measurer) to assess the accuracy of diagnosis.

### 2.3 Statistical analysis

Continuous variables were expressed as median (interquartile range, IQR). The statistical comparisons of continuous variables were analyzed using the Mann‐Whitney *U*‐test, and those of categorical variables were analyzed using the chi‐square test. Univariate Cox proportional hazards analyses to identify risk factors for cardiotoxicity were performed with clinical variables that are generally recognized as affecting cardiotoxicity. In order to consider competing risks of cardiotoxicity and death prior to the onset of cardiotoxicity, cumulative incidence functions of cardiotoxicity and overall survival were calculated using a multivariate model to reveal independent risk factors for cardiotoxicity. Values of *P* < .05 were considered statistically the Aalen‐Johansen estimator. The risk factors with *P* < .1 by univariate cause‐specific Cox regression analyses were entered in significant. All analyses were performed using the Statistical Package for Sciences version 26 (SPSS Inc, Chicago, IL, USA).

## 3 RESULTS

### 3.1 Baseline characteristics

The baseline characteristics of the 229 enrolled patients are shown in Table 1. The median age was 71 (IQR, 63‐77), 53% were male, 64% had DLBCL, 82% received the R‐CHOP regimen, and the median cumulative dose of doxorubicin was 301 (IQR, 239‐392) mg/m^2^. Among DLBCL, the numbers of patients with IVL and PMLBCL were six and two, respectively. Median baseline LVEF and plasma BNP level were 64.6% and 32.2 pg/mL, respectively. A statistically significant difference between patients with and without cardiotoxicity was seen only in history of ischemic heart disease and LVEF at baseline (*P* = .001; Table 1).

**TABLE 1:** Baseline characteristics of 229 patients with lymphoma who received a CHOP‐like regimen

Characteristic	Total n = 229	Cardiotoxicity (+) n = 48 (21%)	Cardiotoxicity (–) n = 181 (79%)	*P* ‐value
Age (year)	71 (63‐77)	73 (66‐78)	70 (62‐77)	.10
Male (n (%))	122 (53%)	27 (56%)	95 (52%)	.64
BMI	21.6 (19.5‐22.0)	21.6 (19.6‐22.0)	21.6 (19.5‐22.0)	.84
R‐CHOP (n (%))	188 (82%)	37 (77%)	151 (83%)	.31
DLBCL (n (%))	146 (64%)	33 (69%)	113 (62%)	.42
Performance status ≥ 2 (n (%))	80 (35%)	20 (42%)	60 (33%)	.27
Doxorubicin dose (mg/m^2^)	301 (239‐392)	307 (244‐393)	300 (237‐388)	.20
Cardiovascular risk factors				
Hypertension (n (%))	81 (35%)	19 (40%)	62 (34%)	.49
Dyslipidemia (n (%))	64 (28%)	14 (29%)	50 (28%)	.83
Diabetes mellitus (n (%))	46 (20%)	12 (25%)	34 (19%)	.34
Smoking (n (%))	90 (39%)	21 (44%)	69 (38%)	.48
History of cardiovascular disease				
Ischemic heart disease (n (%))	10 (4%)	6 (13%)	4 (2%)	.002
Heart failure (n (%))	7 (3%)	2 (4%)	5 (3%)	.62
Atrial fibrillation (n (%))	11 (5%)	3 (6%)	8 (4%)	.60
Cerebral infarction (n (%))	11 (5%)	1 (2%)	10 (6%)	.32
Laboratory measurements				
Hemoglobin (g/dL)	11.8 (10.2‐13.3)	11.9 (10.3‐13.3)	11.6 (10.1‐13.3)	.77
Albumin (mg/dL)	3.6 (2.9‐4.1)	3.7 (2.8‐4.0)	3.6 (2.9‐4.1)	.81
Serum sodium (mEq/L)	140 (137‐142)	139 (137‐141)	140 (138‐142)	.14
eGFR (mL/min/1.73 m^2^)	72.3 (56.8‐85.4)	65.4 (52.4‐82.9)	73.8 (58.0‐87.1)	.13
Total bilirubin (mg/dL)	0.6 (0.5‐0.9)	0.6 (0.5‐0.8)	0.6 (0.5‐0.9)	.82
BNP (pg/mL; n = 198)	32.2 (18.8‐58.7)	43.1 (20.3‐66.7)	29.2 (18.2‐57.1)	.14
Echocardiography				
LVEF (%)	64.6 (59.8‐68.5)	61.3 (58.0‐64.9)	65.0 (60.0‐69.0)	.001
Valvular disease ≥ moderate (n (%))	33 (14%)	8 (17%)	25 (14%)	.60

Abbreviations: BMI, body mass index; BNP, brain natriuretic peptide; DLBCL, diffuse large B‐cell lymphoma; eGFR, estimated glomerular filtrating rate; LVEF, left ventricular ejection fraction.; R‐CHOP, rituximab, cyclophosphamide, doxorubicin, vincristine, and prednisolone.

History of ischemic heart disease includes prior myocardial infarction, angina pectoris, and history of intervension for coronary artery disease.

### 3.2 Cardiac events

**FIGURE 2:**
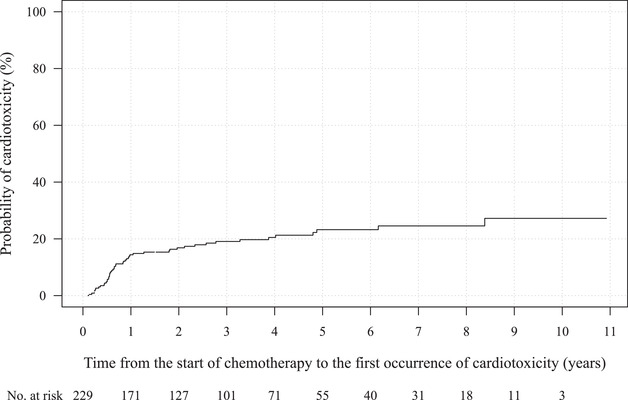
Cumulative incidence curve for cardiotoxicity in all enrolled patients with a consideration of competing risks of cardiotoxicity and death prior to the onset of cardiotoxicity using the Aalen‐Johansen estimator. With a median follow‐up period of 1132 days, 48 patients developed cardiotoxicity and the median time from the start of chemotherapy to the first occurrence of cardiotoxicity was 250 days. Thirty‐three patients (69%) developed cardiotoxicity during the first year from the initiation of chemotherapy

With a median follow‐up period of 1132 days (IQR, 492‐1956), cardiotoxicity was observed in 48 of 229 patients (21%). Median and mean number of follow‐up echocardiographic study were 2 (IQR, 1‐4) and 3.0 (SD, 2.7). Median interval duration of all echocardiographic follow‐up study was 112 days (IQR, 49‐240). Median time to the first occurrence of cardiotoxicity was 250 days (IQR, 185‐704; Figure 2), and cardiotoxicity within the first year after starting a CHOP regimen with or without rituximab was observed in 32 patients (67%). Of these 48 patients with confirmed cardiotoxicity, symptomatic heart failure and cardiovascular death were observed in 30 (13%) and five (2%) patients, respectively (Figure 1). Detailed clinical information on the five patients who developed cardiovascular death is shown in Table S1.

On the other hand, describing except for the prevalence of decline in LVEF or heart failure, new‐onset atrial fibrillation was observed in 25 patients (8 in patients with cardiotoxicity, 17 in patients without cardiotoxicity), consultation for cardiologists due to non‐sustained ventricular tachycardia was performed in 6 patients (4 and 2 in patients with and without cardiotoxicity), and myocardial infarction and angina pectoris developed in two and one patients after chemotherapy, respectively. Sustained ventricular tachycardia was not recorded in all participants, and one patient developed myocardial infarction 3 months after the start of chemotherapy resulting in meeting the criteria of cardiotoxicity.

### 3.3 Risk factors for cardiotoxicity

Univariate Cox proportional hazards analysis showed that older age, history of ischemic heart disease, decreased estimated glomerular filtration ratio, higher log‐BNP, and lower LVEF were significantly associated with the risk of cardiotoxicity (Table 2). Multivariate Cox proportional hazards analysis demonstrated that history of ischemic heart disease (HR, 3.15; 95% CI, 1.17‐8.47; *P* = .023) and baseline LVEF (HR per 10% decrease, 2.47; 95%CI, 1.49‐4.06; *P *< .001) as independent risk factors for cardiotoxicity (Table 2).

**TABLE 2:** Cause‐specific Cox regression analysis of risk factors for cardiotoxicity

	Univariable analysis			Multivariable analysis		
Variable	HR	95% (CI)	*P*‐value	HR	95% (CI)	*P*‐value
Basic data						
Age, per 10‐year increase	1.61	1.18‐2.19	.003	1.22	0.82‐1.82	.32
Male	1.20	0.68‐2.12	.54			
BMI	1.01	0.90‐1.12	.92			
R‐CHOP, versus CHOP	0.55	0.28‐1.08	.083	0.58	0.28‐1.21	.15
DLBCL, versus non‐DLBCL	1.18	0.64‐2.18	.59			
Performance status ≥ 2, versus ≤ 1	1.74	0.98‐3.09	.061	1.51	0.81‐2.82	.19
Doxorubicin dose, per 10 mg/m² increase	1.00	0.97‐1.03	.91			
Cardiovascular risk factors ≥ 2, versus ≤ 1	1.37	0.77‐2.42	.28			
History of ischemic heart disease	5.08	2.15‐12.01	<.001	3.15	1.17‐8.47	.023
Laboratory measurements at baseline						
Hemoglobin (g/dL)	0.98	0.87‐1.10	.71			
Albumin (mg/dL)	0.85	0.59‐1.22	.37			
Serum sodium (mEq/L)	0.96	0.89‐1.03	.23			
eGFR, per 10 mL/min/1.73m^2^ decrease	1.16	1.02‐1.31	.020	1.04	0.90‐1.20	.65
Total bilirubin (mg/dL)	0.98	0.61‐1.57	.93			
Log BNP (pg/mL)*	2.34	1.16‐4.69	.017	1.70	0.79‐3.69	.18
Echocardiography at baseline						
LVEF, per 10% decrease	2.11	1.31‐3.40	.002	2.47	1.49‐4.06	<.001
Valvular disease ≥ moderate, versus ≤ mild	1.43	0.67‐3.06	.36			

Abbreviations: BMI, body mass index; BNP, brain natriuretic peptide; cardiovascular diseases are myocardial infarction, heart failure, and cerebral infarction.; Cardiovascular risk factors are hypertension, dyslipidemia, diabetes mellitus, and history of smoking; CHOP, rituximab, cyclophosphamide, doxorubicin, vincristine, and prednisolone; DLBCL, diffuse large B‐cell lymphoma; eGFR, estimated glomerular filtrating rate; LVEF, left ventricular ejection fraction; R‐CHOP, rituximab, cyclophosphamide, doxorubicin, vincristine, and prednisolone.

History of ischemic heart disease includes prior myocardial infarction, angina pectoris, and history of intervension for coronary artery disease.

* Plasma BNP levels were missing for 31 patients, and thus univariate analysis with BNP as independent variable and multivariable analysis were performed for 198 patients.

**FIGURE 3:**
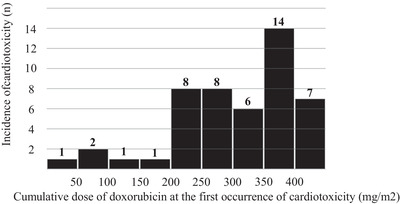
Distribution of the incidence of first occurrence of cardiotoxicity with cumulative dose of doxorubicin. Cardiotoxicity was frequently observed in patients who received more than 200 mg/m^2^

According to the distribution of incidence of cardiotoxicity and cumulative doxorubicin dose, cardiotoxicity was observed even in patients who received 200‐250 mg/m^2^ of doxorubicin (Figure 3).

### 3.4 Incidence of recovery from cardiotoxicity and prognosis after recovery

Among the 48 patients who experienced cardiotoxicity during the follow‐up period, full recovery and partial recovery were observed in nine and 12 patients, respectively; a total of 21 patients (44%) recovered LVEF to normal range (≥50%) after cardiotoxicity occurred. The time from cardiotoxicity to recovery in all 21 patients was 463 (IQR, 96‐677) days; in the nine patients with full recovery, it was 484 days; and in the 12 patients with partial recovery, it was 398 days. Among those patients who achieved recovery, 15 (71%) received anti‐remodeling medication (angiotensin‐converting enzyme inhibitor, angiotensin 2 receptor blocker, beta blocker, or mineralocorticoid receptor antagonist). Details of the medications are shown in Table 3. Two of nine patients (22%) who achieved full recovery and 4 of 12 patients (33%) who achieved partial recovery developed re‐cardiotoxicity. One patient with full recovery had a sudden death, and there were 4 deaths due to heart failure in patients with no recovery (Table 3; Table S1).

**TABLE 3:** Outcomes in 48 lymphoma patients with cardiotoxicity who received a CHOP‐like regimen

Cardiotoxicity (+)	Recovery pattern after cardiotoxicity	Medication at cardiotoxicity; ACEI or ARB, BB, MRA	Duration from cardiotoxicity to recovery, days	Symptom (+) at cardiotoxicity	Re‐cardiotoxicity (+)	Death due to heart failure	Sudden death
n = 48	Full recovery (n = 9)	5 (56%), 6 (67%), 4 (44%)	484 (183‐586)	6 (67%)	2 (22%)	0	1 (11%)
	Partial recovery (n = 12)	5 (42%), 6 (50%), 2 (17%)	398 (73‐820)	8 (67%)	4 (33%)	0	0
	No recovery (n = 27)	9 (33%), 8 (30%), 10 (37%)	–	16 (59%)	–	4 (15%)	0

Abbreviations: ACEI, angiotensin‐converting enzyme inhibitor; ARB, angiotensin 2 receptor blocker; BB, beta blocker; MRA, mineralocorticoid receptor antagonist.

Full recovery was defined as improving to 95% or more of baseline LVEF, and partial recovery was defined as a ≥5% increase in LVEF and LVEF > 50% after development of cardiotoxicity. Re‐cardiotoxicity was defined as meeting the criteria of cardiotoxicity again after partial recovery or full recovery was achieved. The percentiles are for each recovery pattern.

### 3.5 Impact of cardiotoxicity on survival

All death was observed in 78 patients during the observational period. Forty‐eight patients (62%) died due to lymphoma progression. The remaining 30 patients died due to the following causes; chemotherapy‐related (n = 9, 12%), non‐lymphoma cancer (n = 10, 13%), and the other reasons (n = 11, 14%). All‐cause mortality in patients with cardiotoxicity tended to be higher than in patients without cardiotoxicity, but the difference was not significant (log‐rank *P *= .15; Figure S1). There was no significant difference in overall mortality between patients who developed symptomatic heart failure and those who did not. Individual details of patients who developed cardiotoxicity and died from cardiovascular causes are summarized in Table S1.

## 4 DISCUSSION

We chiefly demonstrated that 21% (48/229) of patients with ML who received a first‐line CHOP regimen with or without rituximab developed cardiotoxicity; 13% had symptomatic heart failure, and 2% had cardiovascular death, with a median follow‐up of 3.1 years. This report is the first to assess cardiotoxicity, recovery from cardiotoxicity, and recurrence of cardiotoxicity with a focus on patients with ML who received a CHOP regimen with or without rituximab. In previous studies, the incidence of cardiotoxicity has been reported to be 5.4% to 27% in patients with variable malignant disease who received anthracycline‐containing chemotherapy [17,20, 23,24] (Table S2).

Although cardiotoxicity is seen frequently after use of over 400 mg/m^2^ of doxorubicin and the incidence of cardiotoxicity was dose dependent in previous studies, a recent prospective study showed that even 250 mg/m^2^ of doxorubicin is a risk factor and that there may be no safe dosage without risk of doxorubicin‐induced cardiotoxicity [12, 15]. Here, we observed no significant relationship between cardiotoxicity and cumulative dose of doxorubicin: some patients treated with low‐dose doxorubicin had cardiotoxicity, suggesting that patients who receive 200 mg/m^2^ or more of doxorubicin are potentially at risk for cardiotoxicity. Thus, close monitoring of LVEF using echocardiography and surrogate serological markers such as troponin or BNP or NT‐proBNP may be needed to diagnose cardiotoxicity at an early stage in patients who receive a cumulative dose of doxorubicin of 200 mg/m^2^ or more.

In our study, baseline risk factors of cardiotoxicity were older age, history of ischemic heart disease, renal dysfunction, high BNP level, and lower LVEF within normal limit in the univariate Cox proportional hazards analysis. The analysis showed no significant correlation between general cardiovascular risk factors and cardiotoxicity, partly because our relatively recent participants might have already received adequate treatments for general cardiovascular risk factors. History of ischemic heart disease is a well‐known risk factor for anthracycline‐induced cardiotoxicity in the previous study [25, 26], thus our result supported and strengthened this knowledge. Although the main metabolic pathway of anthracycline is liver, renal dysfunction possibly causes elevated blood concentration of anthracycline, and this may result in cardiotoxicity. Renal dysfunction also results in decreased erythropoietin, which is reported to have a cardioprotective role [27, 28]. In a recent large‐scale prospective study that included some types of chemotherapy regimens for other cancers, lower LVEF at the end of chemotherapy, older age, female sex, family history of coronary artery disease, and higher cumulative anthracycline dose were identified as independent risk factors by multivariate Cox proportional hazard regression with forward stepwise selection [20]. A previous prospective study with a limited number of patients with ML indicated that older age, male sex, radiotherapy, higher cumulative dose of anthracycline, and overweight were the independent risk factors of cardiotoxicity by multivariate logistic regression analyses [24], however, in which the strict data of baseline LVEF was not available. Change of contractility of LV can be influenced by lots of clinical factors, however only history of ischemic heart disease and LVEF at baseline were the independent risk factors for cardiotoxicity assessed with many factors for which analysis were desirable in our study, and we speculate that cardiac condition before chemotherapy is the most relevant influence on cardiotoxicity in our cohort, even in patients whose LVEF is within normal limits.

In that large‐scale prospective study, 98% of anthracycline‐induced cardiotoxicity was observed within 1 year after the start of chemotherapy in a heterogeneous patient population with variable primary diseases, in particular half or more of the patients had breast cancer, and chemotherapy regimens [20]. In our study, 67% of cardiotoxicity was observed within 1 year from the start of chemotherapy, with the remaining 33% observed after 1 year. Possible reasons for the difference in our results include the following: our participants were limited to patients with ML who received a CHOP regimen with or without rituximab, basic characteristics such as baseline age and renal function were different, and it might be associated with the varied timing of follow‐up echocardiography in some patients.

We also analyzed changes in cardiac function over time after the development of cardiotoxicity. Partial recovery or full recovery were seen in 43% of patients with cardiotoxicity. We gathered serial data on cardiac contractions after recovery from cardiotoxicity. Two of nine patients (22%) with full recovery experienced a recurrence of cardiotoxicity, although four of 12 patients (33%) with partial recovery developed recurrent cardiotoxicity, even though some had not only discontinued chemotherapy but also received treatments for cardiotoxicity. Anthracycline‐induced cardiomyopathy is recognized as type 1 myocardial damage that is considered cumulative and permanent [2, 29, 30]. Even if medical therapy for heart failure increases left ventricular contraction, myocardial tissue damage persists and we need watchful follow‐up of patients with full or partial recovery. It is possible that the patients who did not experience a recurrence of cardiotoxicity had not only irreversible myocardial damage due to anthracycline, but also reversible changes such as those due to acute inflammation. Previous research has described early detection and prompt administration of anti‐remodeling therapy as crucially important to improving the cardiac systolic function of patients with anthracycline‐induced cardiotoxicity [31, 32, 33]. Therefore, we must start therapy for cardiotoxicity as early as possible to limit irreversible myocardial injury to a minimum.

Most cardiovascular deaths in this study were observed in patients who never achieved any degree of recovery after cardiotoxicity. This finding indicates the necessity of maximizing efforts to improve LVEF, once LVEF decline is observed. Furthermore, it is very important to diagnose the cardiotoxicity at an early stage in high‐risk patients. Some biomarkers [34, 35, 36] and decreased global longitudinal strain [37, 38] have been reported to be useful in the early diagnosis of cardiotoxicity. Furthermore, early diagnosis and therapy for cardiotoxicity have been reported to be associated with better outcome [31, 32, 33]. Importantly, primary preventive use of a beta blocker was examined in a recent study [39]. With appropriate consideration of cost‐benefit ratios and risks of administration, further development of preventive strategies is expected, especially for patients at high risk for cardiotoxicity.

Our study has some limitations. First, it was a single‐center, retrospective study and the number of enrolled patients was limited. Second, patients who did not undergo baseline or follow‐up echocardiography were excluded, and the timing of follow‐up echocardiography was relatively varied depending on the attending physician. The incidence of cardiotoxicity might be overestimated. Third, our study had no data on global longitudinal strain, which is considered to be an early indicator of cardiotoxicity.

## 5 CONCLUSION

In patients with ML who received a CHOP regimen with or without rituximab, 21% developed cardiotoxicity, 13% had symptomatic heart failure, and 2% died from cardiovascular causes. Our multivariate analysis showed that only baseline LVEF and history of ischemic heart disease were the independent risk factors for cardiotoxicity, indicating cardiac condition before chemotherapy is the most relevant for developing cardiotoxicity. Forty‐four percent of patients who developed cardiotoxicity achieved LVEF recovery; however, 29% of patients with recovery from cardiotoxicity developed LVEF decline again. Watchful follow‐up and continuous management for LV dysfunction must be required in patients with cardiotoxicity, even after LVEF recovery.

## AUTHOR CONTRIBUTIONS

Takafumi Nakayama, Yoshiko Oshima, Junki Yamamoto, and Shigeru Kusumoto designed and performed the research. Takafumi Nakayama and Yoshiko Oshima wrote the paper, and Shigeru Kusumoto oversaw the paper and the whole research. Satoshi Osaga supervised the statistical analysis in this research. Yoshiko Oshima, Shigeru Kusumoto, Haruna Fujinami, Takaki Kikuchi, Tomotaka Suzuki, Haruhito Totani, Shiori Kinoshita, Tomoko Narita, Asahi Ito, Masaki Ri, Hirokazu Komatsu, and Shinsuke Iida collected the clinical data. Takafumi Nakayama, Shigeru Kusumoto, Kazuaki Wakami, Toshiaki Goto, Tomonori Sugiura, Yoshihiro Seo, and Nobuyuki Ohte revised the paper critically. All authors approved the submission of the paper of final version.

## CONFLICT OF INTEREST

The authors declare no conflict of interest.

## Supporting information

Supporting information.
Figure S1. Kaplan‐Meier curves for all‐cause death according to cardiotoxicity. Patients who experienced cardiotoxicity had a relatively low survival rate compared with patients without cardiotoxicity, but there was no statistically significant difference (log‐rank p = 0.15).
Table S1 Summary of 5 patients with cardiotoxicity who died due to cardiovascular diseases.
Table S2 Summary of studies regarding anthracycline‐induced cardiotoxicity in patients with malignant lymphoma.
