# Supplementary material for: Clinical features of anthracycline‐induced cardiotoxicity in patients with malignant lymphoma who received a CHOP regimen with or without rituximab: A single‐center, retrospective observational study
Source: EJHaem. 2020 Oct 3;1(2):498–506. doi: 10.1002/jha2.110 (PMC9176145; doi:10.1002/jha2.110)
Supplement: Supplementary file 1 — Supporting information. Figure S1. Kaplan‐Meier curves for all‐cause death according to cardiotoxicity. Patients who experienced cardiotoxicity had a relatively low survival rate compared with patients without cardiotoxicity, but there was no statistically significant difference (log‐rank p = 0.15). Table S1 Summary of 5 patients with cardiotoxicity who died due to cardiovascular diseases. Table S2 Summary of studies regarding anthracycline‐induced cardiotoxicity in patients with malignant lymphoma. [file JHA2-1-498-s001.pdf]

SUPPORTING INFORMATION

Title:

Clinical features of anthracycline-induced cardiotoxicity in patients with malignant lymphoma who received a CHOP regimen with or without rituximab: a single-center, retrospective observational study

Authors:

Takafumi Nakayama[1*], Yoshiko Oshima[2*], Shigeru Kusumoto[2], Junki Yamamoto[1], Satoshi Osaga[3], Haruna Fujinami[2], Takaki Kikuchi[2], Tomotaka Suzuki[2], Haruhito Totani[2], Shiori Kinoshita[2], Tomoko Narita[2], Asahi Ito[2], Masaki Ri[2], Hirokazu Komatsu[2], Kazuaki Wakami[1], Toshihiko Goto[1], Tomonori Sugiura[1], Yoshihiro Seo[1], Nobuyuki Ohte[1], Shinsuke Iida[2]

Institutions:

[1]Department of Cardiology, Nagoya City University Graduate School of Medical Sciences

[2]Department of Hematology and Oncology, Nagoya City University Graduate School of Medical Sciences

[3]Clinical Research Management Center, Nagoya City University Hospital

*Drs Nakayama and Oshima contributed equally to this work.

Correspondence:

Shigeru Kusumoto, MD, PhD.

Department of Hematology and Oncology, Nagoya City University Graduate School of Medical Sciences

1-Kawasumi, Mizuho-cho, Mizuho-ku, Nagoya, 467-8601, Japan

Tel.: +81-(0)52-853-8737

Fax: +81-(0)52-853-8740

E-mail: skusumot@med.nagoya-cu.ac.jp

Figure S1

Kaplan-Meier curves for all-cause death according to cardiotoxicity. Patients who experienced cardiotoxicity had a relatively low survival rate compared with patients without cardiotoxicity, but there was no statistically significant difference (log-rank P = 0.15).

Table S1

Summary of 5 patients with cardiotoxicity who died due to cardiovascular diseases.

Table S2

Summary of studies regarding anthracycline-induced cardiotoxicity in patients with malignant lymphoma.

Figure S1

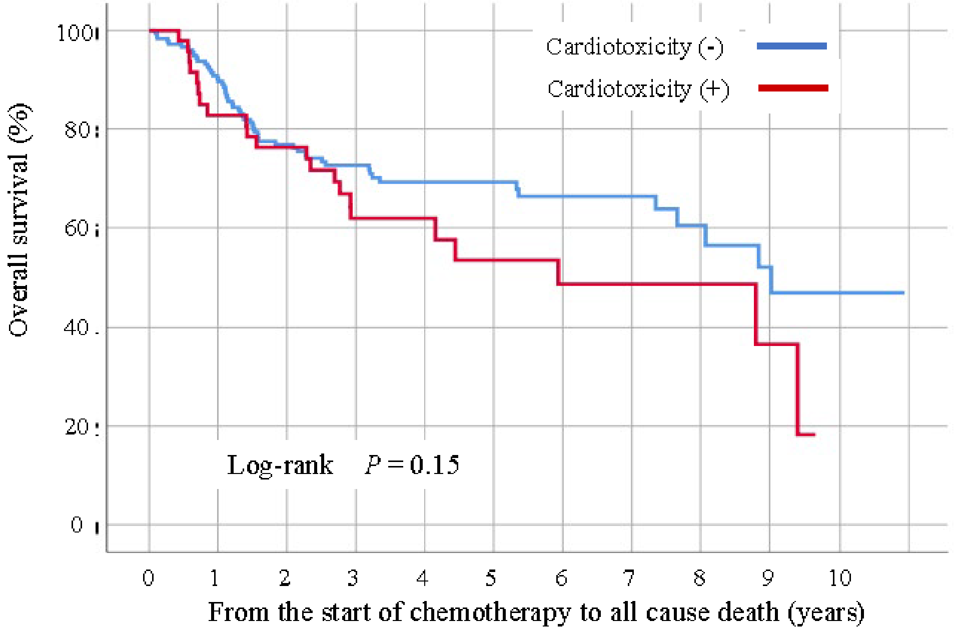

No. at risk

Cardiotoxicity (-)	181	154	115	94	65	52	37	28	16	10
Cardiotoxicity (+)	48	38	35	25	16	13	10	9	7	3

Table S1. Summary of 5 patients with cardiotoxicity who died due to cardiovascular diseases

Case No.	Age	Sex	Lymphoma type	Chemotherapy regimen	Cumulative dose of doxorubicin (mg/m²)	LVEF at baseline (%)	LVEF at cardiotoxicity (%)	Time from the start of chemotherapy to cardiotoxicity (days)	Cause of death
1	73	F	DLBCL	R-CHOP	247	66.2	32.6	94	Heart failure
2	75	F	FL	R-CHOP	300	61.3	47.0	113	Heart failure
3	83	M	DLBCL	R-CHOP	239	59.9	33.7	164	Sudden death
4	81	F	DLBCL	R-CHOP	386	58.3	33.2	238	Heart failure
5	56	M	AITL	CHOP	400	65.2	33.4	853	Heart failure

Abbreviations: M, male; F, female; DLBCL, diffuse large B-cell lymphoma; FL, follicular lymphoma; AITL, angioimmunoblastic T-cell lymphoma; R-CHOP, rituximab, cyclophosphamide, doxorubicin, vincristine, and prednisolone; CHOP, rituximab, cyclophosphamide, doxorubicin, vincristine, and prednisolone

Table S2. Summary of studies regarding anthracycline-induced cardiotoxicity in patients with malignant lymphoma

Study no., First author/reference	Subjects' cancer type(s)	Number of subjects	Study design	Definition of cardiotoxicity	Cumulative dose of anthracycline	Incidence of cardiotoxicity	Symptomatic heart failure	Cardiovascular death
1, Cardinale D *et al*, Circulation, 2015	Breast cancer (51%), non-Hodgkin lymphoma (28%), etc.	2,625	Prospective, multi-center	Reduction in LVEF ≥ 10% and LVEF < 50%	-	9% (226/2625)	NYHA3 or 4 in 19%	0.2% (6/2625)
2, Hequet O *et al*, J Clin Oncol. 2004	Non-Hodgkin lymphoma and Hodgkin lymphoma	141	Prospective, single-center	Fractional shortening < 25%	300 mg/m^2	27.6% (39/141)	0.7% (1/141)	-
3, Dlugosz-Danecka M *et al*, Chemotherapy. 2018	Non-Hodgkin lymphoma	97	Retrospective and prospective, single-center	Reduction in LVEF ≥ 10% and LVEF < 50%	300 mg/m^2	8.2% (8/97)	16.5% (16/97)	9.3% (9/97)
4, Bosch X *et al*, J Am Coll Cardiol. 2013	Acute leukemia, Hodgkin and non-Hodgkin lymphoma, multiple myeloma	37	Prospective, single-center	Reduction in LVEF ≥ 10% and LVEF < 50%	241 mg/m^2	5.4% (2/37)	-	-
Current study	Non-Hodgkin lymphoma	229	Retrospective, single-center	Reduction in LVEF ≥ 10% and LVEF < 50%	301 mg/m^2	21% (48/229)	13% (30/229)	2% (5/244)

Abbreviations: LVEF, left ventricular ejection fraction; NYHA, New York Heart Association class
